# Supplementary material for: Vernonia polysphaera Baker: Anti-inflammatory activity in vivo and inhibitory effect in LPS-stimulated RAW 264.7 cells
Source: PLoS One. 2019 Dec 12;14(12):e0225275. doi: 10.1371/journal.pone.0225275 (PMC6907817; doi:10.1371/journal.pone.0225275)
Supplement: S1 Table — (DOCX) [file pone.0225275.s001.docx]

**S1 Table. Mast cells quantification.** Cell counting under light microscopy of histological analysis in edema of animals inoculated with 30µL of λ-carrageenan 1%, and treated with 100µL *Vernonia polysphaera* hydroalcoholic extract by gavage or with dexamethasone 5mg/kg via the intramuscular route

| Group | λ-carrageenan | Dose (mg/kg) | Mast cells |
| --- | --- | --- | --- |
| Control | - | - | 12.83 ± 8.005 |
| PBS | + | - | 57.67 ± 12.29# |
| *Vernonia polysphaera* extract | + | 50 | 59.13 ± 14.01 |
|  | + | 250 | 44.00 ± 14.14 |
|  | + | 500 | 39.33 ± 12.33 |
| Dexamethasone | + | 5 | 18.83 ± 10.30* |

Data represents mean ± standard deviation of mast cells in five fields selected for counting under a light microscope and are representative of three independent experiments carried out in quintuplicate. #p<0.001 compared with control group; *p=0.014 compared with PBS group, after Kruskal-Wallis followed by Dunn’s multiple comparisons test.
