# Supplementary material for: Vernonia polysphaera Baker: Anti-inflammatory activity in vivo and inhibitory effect in LPS-stimulated RAW 264.7 cells
Source: PLoS One. 2019 Dec 12;14(12):e0225275. doi: 10.1371/journal.pone.0225275 (PMC6907817; doi:10.1371/journal.pone.0225275)
Supplement: S2 Table — (DOCX) [file pone.0225275.s002.docx]

**S2 Table. Mast cells quantification.** Cell counting under light microscopy of histological analysis in edema of animals inoculated with 30µL of λ-carrageenan 1%, and treated with 100µL *Vernonia polysphaera* hydroalcoholic extract by gavage or with dexamethasone 5mg/kg via the intramuscular route

| Group | λ-carrageenan | Dose (mg/kg) | Cells/mL | Leukocytes/mm^3^ | IL-1β (pg/mL) | IL-6 (pg/mL) | TNF-α (pg/mL) | PGE2 (pg/mL) |
| --- | --- | --- | --- | --- | --- | --- | --- | --- |
| Control | - | - | 28083 ± 5795 | 7750 ± 1179 | 26.28 ± 14.69 | 38.93 ± 27.61 | 103.2 ± 56.82 | 960.0 ± 190.9 |
| PBS | + | - | 45000 ± 2817# | 22720 ± 4079# | 468.2 ± 82.29# | 1552 ± 152.3# | 528.4 ± 50.03# | 2150 ± 63.64# |
| *Vernonia polysphaera* extract | + | 50 | 34750 ± 1732 | 22410 ± 5049 | 339.4 ± 29.82 | 1452 ± 131.4 | 419.4 ± 18.13 | 1990 ± 190.9 |
|  | + | 250 | 22083 ± 6948*** | 17230 ± 6851 | 278.9 ± 22.08* | 1199 ± 132.3* | 370.1 ± 74.35 | 1920 ± 63.64 |
|  | + | 500 | 21750 ± 1521*** | 10420 ± 5191* | 174.0 ± 12.66** | 1061 ± 57.09*** | 323.8 ± 63.85* | 495.0 ± 396.0** |
| Dexamethasone | + | 5 | 19500 ± 2291*** | 5300 ± 2386*** | 78.56 ± 23.90*** | 274.0 ± 72.75*** | 120.1 ± 54.98*** | 345.0 ± 98.99*** |

Data represents mean ± standard deviation of mast cells in five fields selected for counting under a light microscope and are representative of three independent experiments carried out in quintuplicate. #p<0.001 compared with control group; *p=0.014 compared with PBS group, after Kruskal-Wallis followed by Dunn’s multiple comparisons test.
