# Supplementary material for: Vernonia polysphaera Baker: Anti-inflammatory activity in vivo and inhibitory effect in LPS-stimulated RAW 264.7 cells
Source: PLoS One. 2019 Dec 12;14(12):e0225275. doi: 10.1371/journal.pone.0225275 (PMC6907817; doi:10.1371/journal.pone.0225275)
Supplement: S3 Table — (DOCX) [file pone.0225275.s003.docx]

**S3 Table. Proinflammatory factors in RAW 264.7 cells stimulated by LPS and treated with *Vernonia polysphaera* extract.** Levels of nitrite, proinflammatory cytokines and PGE-2 in culture supernatants, and mRNA expression of COX-2 in cell culture treated with *V. polysphaera* (10, 50 or 100μg/mL) or dexamethasone, 100 μM

| Group | LPS | (µg/mL) | NaNO_2_ (µM) | IL-1β (pg/mL) | IL-6 (pg/mL) | TNF-α (pg/mL) | PGE2 (pg/mL) | COX-2/B2M (RQ) |
| --- | --- | --- | --- | --- | --- | --- | --- | --- |
| Control | - | - | 5.042 ± 0.937 | 36.25 ± 10.49 | 3.212 ± 1.819 | 82.88 ± 38.01 | 155.0 ± 183.8 | 1.001 ± 0.045 |
| PBS | + | - | 41.86 ± 3.096# | 142.9 ± 37.72# | 288.4 ± 90.57# | 478.7 ± 112.4# | 3212 ± 118.5# | 4.620 ± 0.481# |
| *Vernonia polysphaera* extract | + | 10 | 45.62 ± 3.298 | 107.1 ± 15.60 | 261.8 ± 112.1 | 293.9 ± 63.82 | 3052 ± 190.4 | 2.098 ± 0.349 |
|  | + | 50 | 31.28 ± 2.194*** | 67.78 ± 30.97** | 167.4 ± 36.47** | 200.1 ± 58.99** | 2443 ± 118.7*** | 1.600 ± 0.051 |
|  | + | 100 | 14.89 ± 3.268*** | 30.00 ± 10.09*** | 19.52 ± 8.444*** | 172.5 ± 19.73** | 1480 ± 137.7*** | 1.329 ± 0.169** |
| Dexamethasone | + | 100µM | 23.25 ± 2.556*** | 50.83 ± 5.892*** | 51.25 ± 5.845*** | 188.4 ± 19.62** | 440 ± 91.92*** | 1.455 ± 0.048** |

Data are representative of two independent experiments performed at least in quadruplicate. #p <0.001 compared with the group without stimulation or treatment; **p<0.01, ***p<0.001 compared with the stimulated and untreated group after Kruskal-Wallis analysis followed by Dunn’s multiple comparisons test.
