# Supplementary material for: Vernonia polysphaera Baker: Anti-inflammatory activity in vivo and inhibitory effect in LPS-stimulated RAW 264.7 cells
Source: PLoS One. 2019 Dec 12;14(12):e0225275. doi: 10.1371/journal.pone.0225275 (PMC6907817; doi:10.1371/journal.pone.0225275)
Supplement: S4 Table — (DOCX) [file pone.0225275.s004.docx]

**S4 Table. Proinflammatory transcriptional factor expression in RAW 264.7 cells stimulated by LPS and treated with *Vernonia polysphaera* hydroalcoholic extract.** Relative quantification (RQ) of NF-κB1, NF-κB2, RelA and RelB expression in cell culture treated with *V. polysphaera* (10, 50 or 100μg/mL) or dexamethasone, 100 μM

| Group | LPS | (µg/mL) | NF-κB1/B2M | NF-κB2/B2M | RelA/B2M | RelB/B2M |
| --- | --- | --- | --- | --- | --- | --- |
| Control | - | - | 1.017 ± 0.040 | 1.045 ± 0.049 | 1.025 ± 0.028 | 1.023 ± 0.036 |
| PBS | + | - | 2.971 ± 0.105 | 2.965 ± 0.336 | 4.049 ± 1.071 | 2.190 ± 0.124 |
| *Vernonia polysphaera* extract | + | 10 | 3.571 ± 0.058 | 3.313 ± 0.046 | 3.512 ± 0.500 | 2.348 ± 0.252 |
|  | + | 50 | 0.447 ± 0.136 | 0.333 ± 0.067** | 0.784 ± 0.388 | 0.349 ± 0.183* |
|  | + | 100 | 0.354 ± 0.095** | 0.289 ± 0.101** | 0.703 ± 0.305* | 0.327 ± 0.034* |
| Dexamethasone | + | 100µM | 0.531 ± 0.011 | 0.397 ± 0.037 | 0.503 ± 0.021* | 0.307 ± 0.030** |

Data are representative of two independent experiments performed at least in quadruplicate. #p <0.001 compared with the group without stimulation and without treatment. p<0.05, **p<0.01 compared with the group stimulated with untreated LPS after Kruskal-Wallis analysis followed by Dunn’s multiple comparisons test.
