# Supplementary material for: Vernonia polysphaera Baker: Anti-inflammatory activity in vivo and inhibitory effect in LPS-stimulated RAW 264.7 cells
Source: PLoS One. 2019 Dec 12;14(12):e0225275. doi: 10.1371/journal.pone.0225275 (PMC6907817; doi:10.1371/journal.pone.0225275)
Supplement: Attachment — Submitted filename: Response to Reviewers Plos One.docx [file pone.0225275.s005.docx]

Dear Editor of PLOS ONE

Partha Mukhopadhyay, Ph.D.

Thank you very much for all your work on our manuscript and we would like to thank you and the referees for the valuable suggestions. We have addressed all the points raised by the referees; we have marked the relevant corrections in the current version of the manuscript. According to those helpful comments, we made a careful revision on our manuscript “***Vernonia polysphaera* Baker: anti-inflammatory activity in vivo and inhibitory effect in LPS-stimulated RAW 264.7 cells**” (PONE-D-19-24305).

Now we will submit you a revised manuscript version, with marked alterations, and our point to point answers to the comments. The major responses are listed below. We manage to minimize all kinds of errors and obscurities in order to make it understood without difficulty.

**Reviewer #1** *In the present study, the authors evaluated the anti-inflammatory effects of hydroalcoholic extract of a plant Vernonia polysphaera Baker in mouse peripheral and central inflammatory lesion models. The authors performed comprehensive analysis in both in vivo and in vitro effects of said plant extract. The experiments are carried out with balanced designs and results are properly interpreted. There are a few minor details that requires addressing: Methods: Please indicate type of culture medium used to reconstitute the extract for in vitro and in vivo use (Line 101). The extract was prepared in 3 different ways: in DMSO, in culture medium and/or in PBS. For culture medium and PBS prepared extract, which was used in what experiments? I suggest including the info in respective paragraphs of experiments. Same goes when mentioning control or standard reagents for measurements, e.g. nitrite standards prepared in culture medium if that is the case. Table 1: include two-tailed p-values for each extract-treated group to control group.*

**Answer** The detailed procedure of dilution extracts was described in Methods section (Lines 100-105). Reference drugs and standard reagents dilutions were also added to the manuscript in respective paragraphs of experiments (Lines 142; 181; 188; 194).

Two-tailed p-values for extract-treated group when compared to control group were added to manuscript (Lines 240-241).

**Reviewer #2** *In this manuscript, the authors described the action by which organic extracts from Vernonia polysphaera Baker reduces inflammation both in vivo and in vitro. The study was well presented and performed with care. I have the following minor points, mostly relating to the model presented in figure 6.*

*1) Does Vernonia polysphaera extracts reduce both NFkB activation and pro-inflammatory cytokine production by independent actions as shown in Figure 6? Or is the reduction of pro-inflammatory cytokine a result of reduced NFkB activity?*

*2) Does Vernonia polysphaera extracts inhibit NFkB signal by interfering with the processing of NFkB precursor or its generation? Ideally, a simple set of experiments that should be included is western blotting for both the precursor and processed NFkB subunits in Vernonia polysphaera treated vs. non-treated groups.*

*I am a traditionalist concerning quantifying signaling molecules using PCR method and would like to see measurable protein changes. I leave it up to the editor to decide whether this condition needs to be met.*

**Answer**

1) That’s a good point. We believe that *V. polysphaera* extracts reduce NFkB activation preventing DNA-NF-κB binding, and thus suppressing the transcription of COX-2, and consequent PGE-2 production, as well as inducing low levels of inflammatory mediators such as NO, TNF-α, IL-6, IL-1β, as described in caption of Figure 6. We modified the figure to clarify this point.
2) We agree with the reviewer’s comment concerning western blotting analyzes to verify the precursor and processed NFkB subunits alterations induced by *V. polysphaera* treatment. Thus, as response to the reviewer’s comment, we have presented a new approach about this question. However, the overall set of results that we demonstrated with the in vitro experiments allows us to show that *V. polysphaera* extract inhibits mRNA transcription of the NFkB subunits, which is directly linked to the other consistent results (COX-2, PGE2, cytokines and NO low levels) related to inhibition of NFkB activation. Still, to perform a new set of in vitro experiments, as we no longer have the extract available due to the large amount used in the in vivo experiments, we would need to perform a new extraction and new chemical characterization, which would take time, effort and money that unfortunately we do not have it at the moment. We strongly believe that the description of the results presented allow us to conclude that *V. polysphaera* extract has anti-inflammatory effects in vivo and in vitro.

Sincerely,

Fernando Almeida-Souza, PhD (corresponding author – fernandoalsouza@gmail.com)

Instituto Oswaldo Cruz/Fundação Oswaldo Cruz, Laboratório de Imunomodulação e Protozoologia, Pavilhão Carlos Chagas, Sala 213, Manguinhos, Rio de Janeiro, 21040-360.

Tel: 55 - 21 2562 1859/ FAX : 55 - 21 2562 1861/ Cel: 55 - 21 - 98023 2749
